# Fragmentation of SIV-gag Vaccine Induces Broader T Cell Responses

**DOI:** 10.1371/journal.pone.0048038

**Published:** 2012-10-31

**Authors:** Adel Benlahrech, Andrea Meiser, Shanthi Herath, Timos Papagatsias, Takis Athanasopoulos, Fucheng Li, Steve Self, Veronique Bachy, Catherine Hervouet, Karen Logan, Linda Klavinskis, George Dickson, Steven Patterson

**Affiliations:** 1 Department of Medicine, Imperial College London, Chelsea and Westminster Hospital, London, United Kingdom; 2 School of Biological Science, Royal Holloway University of London, Egham, United Kingdom; 3 Fred Hutchinson Cancer Research Center, Seattle, Washington, United States of America; 4 Peter Gorer Department of Immunobiology, Guys Hospital, Kings College London, London, United Kingdom; 5 Department of Immunology, Imperial College London, London, United Kingdom; National Institute of Infectious Diseases, Japan

## Abstract

**Background:**

High mutation rates of human immunodeficiency virus (HIV) allows escape from T cell recognition preventing development of effective T cell vaccines. Vaccines that induce diverse T cell immune responses would help overcome this problem. Using SIV gag as a model vaccine, we investigated two approaches to increase the breadth of the CD8 T cell response. Namely, fusion of vaccine genes to ubiquitin to target the proteasome and increase levels of MHC class I peptide complexes and gene fragmentation to overcome competition between epitopes for presentation and recognition.

**Methodology/Principal Findings:**

Three vaccines were compared: full-length unmodified SIV-mac239 gag, full-length gag fused at the N-terminus to ubiquitin and 7 gag fragments of equal size spanning the whole of gag with ubiquitin-fused to the N-terminus of each fragment. Genes were cloned into a replication defective adenovirus vector and immunogenicity assessed in an *in vitro* human priming system. The breadth of the CD8 T cell response, defined by the number of distinct epitopes, was assessed by IFN-γ-ELISPOT and memory phenotype and cytokine production evaluated by flow cytometry. We observed an increase of two- to six-fold in the number of epitopes recognised in the ubiquitin-fused fragments compared to the ubiquitin-fused full-length gag. In contrast, although proteasomal targeting was achieved, there was a marked reduction in the number of epitopes recognised in the ubiquitin-fused full-length gag compared to the full-length unmodified gene, but there were no differences in the number of epitope responses induced by non-ubiquitinated full-length gag and the ubiquitin-fused mini genes. Fragmentation and ubiquitination did not affect T cell memory differentiation and polyfunctionality, though most responses were directed against the Ad5 vector.

**Conclusion/Significance:**

Fragmentation but not fusion with ubiquitin increases the breadth of the CD8 T vaccine response against SIV-mac239 gag. Thus gene fragmentation of HIV vaccines may maximise responses.

## Introduction

For human immunodeficiency virus (HIV) and its simian counterpart (SIV), the role of cytotoxic T lymphocyte (CTL) responses in controlling viral replication and disease progression has been highlighted in numerous studies (reviewed in [1], [2]). CD8 T cells eliminate HIV-1 infected CD4 T cells *in vitro* [3], and their depletion *in vivo* correlates with impaired viral control in acute and chronic SIV infection [4]–[6]. The association with certain HLA class I alleles and delayed disease progression [7] and the emergence of CTL escape mutants in HIV and SIV infections argues for a protective role for CD8 T cells [8], [9]. Furthermore strong and polyfunctional CTL responses seen in long-term non-progressors [10], [11] and exposed seronegative individuals have been associated with improved control of viral replication [12]. For these reasons attention has focused on developing HIV vaccines that induce CD8 T cell responses, but to date such vaccines have had limited or no success in human or non human primate vaccine trials (reviewed in [13]).

One reason for the failure of CD8 T cell inducing HIV/SIV vaccines to induce protective immunity may reflect the limited breadth of the response. Primary CD8 T cell responses to pathogens, including HIV and SIV, and vaccines tend to be focused on very few epitopes, with most of the response directed against a single dominant epitope and lesser responses against one or two subdominant epitopes [14], [15]. The high mutation rate of HIV/SIV enables the virus to escape rapidly from immune recognition and is exacerbated because many responses are generated against the less conserved domains of the virus [16]. The rules governing the immunodominance hierarchy are the subject of debate, though in broad terms, peptide generation, peptide stability and peptide binding affinity to MHC class I molecules as well as their abundance on the surface of antigen presenting cells (APC), particularly dendritic cells (DC) [17], are important (reviewed in [18]). Competition, which can be at two levels, is also thought to play an important role in determining the immunodominant epitope hierarchy. First, in the endoplasmic reticulum different peptides compete for binding to MHC class I molecules, determined to a large extent by peptide concentration and binding affinity. Second, CD8 T cells recognising different epitopes compete for access to their cognate epitope on the APC surface or for factors produced by APCs, such that a successful CD8 T cell will determine whether a particular epitope becomes immunodominant [19]–[22]. Factors determining the outcome of competition on the APC surface include the affinity of the T cell receptor (TCR) and the number of specific peptide MHC class I complexes [20]–[24]. The net outcome of these events may be to generate a narrow CTL response that is targeted to a small number of pathogen epitopes.

One strategy to overcome immune escape and enable virus control is to develop vaccines that induce CD8 T cells against multiple epitopes. Several vaccine strategies have been described to overcome immunodominance and stimulate a broader CD8 T cell response. For example, a polyvalent mosaic immunogen approach [25] is based on mosaic proteins assembled from fragments of natural sequences via an in silico method to resemble natural proteins, but maximizing the coverage of potential T-cell epitopes [26], [27]. Recent studies in non-human primates demonstrated mosaic vaccines broaden the range of recognizable epitopes and increase the response to high-frequency epitopic variants [26], [27]. Unfortunately, the data suggests that despite increasing antigenic ‘depth’ against epitopic variants, mosaic immunogens do not lead to a significant increase in the number of distinct epitope regions recognised. Hence, an alternative approach is to exploit the attachment of ubiquitin to a vaccine gene thereby forcing their targeting to the proteasome for degradation and it is through this pathway that peptides are generated for presentation by MHC class I molecules. Thus, the vaccine gene is subjected to a higher rate of proteasomal degradation and consequently higher levels of vaccine peptide-MHC class I complexes on the cell surface [28]–[33]. A further approach to broadening CTL responses is to limit antigenic competition, both in the endoplasmic reticulum and on the surface of the APC, by fragmenting the vaccine gene and cloning each fragment into a separate vector [34], [35]. The aim is to express each fragment in a different APC, facilitated by different injection sites, thus preventing competition between epitopes and increasing the breadth of the CD8 T cell response [22], [24]. By this strategy a strong response could be induced against otherwise subdominant epitopes which can confer protection as shown by the observation that vaccination of mice with a subdominant influenza epitope can protect from challenge [36].

Although both ubiquitination and gene fragmentation have been described previously in the context of vaccination in mice [30], [32], [34], [35], here we evaluated the combined strategies in a human system. Unmodified full-length vaccine gene was compared with a full-length gene fused to ubiquitin at the N-terminus and a fragmented gene with the ubiquitin gene fused to the N-terminus of each fragment. We compared the breadth of the T cell response induced by these different vaccine constructs cloned in a replication defective adenovirus type 5 vector (Ad5). To test the constructs, we designed an in-vitro experimental system whereby human monocyte-derived DC (moDC) were transduced with the Ad5 vaccine vectors, and co-cultured with autologous naїve T cells, and antigen specific responses expanded by boosting with autologous vector pulsed moDC on a weekly basis. This system, previously described by Charles Rinaldo and colleagues, has been used to assess HIV-1 immunogens in the context of prophylactic and therapeutic immunisation [37]–[39]. In this study, we chose SIV gag as the test antigen as future experiments are planned with these vaccines in the SIV/macaque model.

## Materials and Methods

### Genes and fragmentation strategy

SIV-mac251 codon optimised gag gene was used as a model gene to study whether ubiquitination and gene fragmentation diversify the T cell response. An *in vitro* priming system employing human T cells and dendritic cells (DC) was developed to test these strategies. The choice of an SIV rather than an HIV gene in this context was made to allow for direct comparison between this *in vitro* study and planned future investigations *in vivo*. SIV-mac251 gag sequence was divided into 7 fragments (MF-1-MF-7) of similar length (69–92 amino-acids) overlapping by 10 amino-acids (Figure S1A and B) and cloned into a replication-deficient E1 and E3-deleted adenovirus type 5 vector (Ad5).

Recombinant Ad5 vectors were generated by insertion of mRNA sequence optimised Ub(G76V)-fused HA-tagged SIV gag genes (GeneArt, Germany) into Ad5 shuttle vectors (pShuttle(−) from Capital Biosciences USA) and ligation with Ad5 vector backbones, followed by large-scale production in packaging cell lines and virus purification (Vector Biolabs, USA). A schematic representation of the different vector constructs used in this study is shown in Figure S1C. All viruses used in this study were aliquoted and stored at −80°C until further use.

### Blood samples

Blood leukocyte cones, products of leukapheresis, were purchased from the North London Blood Transfusion Service (London, UK). Peripheral blood mononuclear cells (PBMC) were isolated by centrifugation over Lymphoprep™ 1077 (PAA Laboratories GmbH, UK) and then separated into a low-density fraction enriched for monocytes and a high-density fraction enriched in T lymphocytes by further centrifugation over a 50% Percoll gradient (Sigma-Aldrich, UK). Both fractions were aliquoted, frozen, and stored in liquid nitrogen until further use.

### Generation of dendritic cells

Human monocyte-derived DC (moDC) were generated as previously described by Sallusto and Lanzavechia [40] with slight modification. In brief, monocytes were isolated from the low density Percoll fraction using CD14 microbeads (Miltenyi Biotec, Germany) according to the manufacturer's protocol. Cells were then cultured for 7 days in complete media (RPMI-1640, PAA laboratories, UK) supplemented with 10% fetal bovine serum (Sigma Aldrich, UK), 1% L-glutamine, and 1% penicillin/streptomycin [both from PAA laboratories]). IL-4 and GM-CSF (both at 100 ng/ml each, R&D Systems, UK) were added to the cultures at days 0, 2, 4, and 6. The resulting immature DC were washed twice and re-suspended in RPMI-1640 in the absence of serum prior to transduction with Ad5 vectors. Note that for each donor, DC were generated on a weekly basis from frozen low density Percoll fractions to boost expanded T cell cultures for at least 3 to 4 weeks (Figure S1A).

### DC transduction with Ad5 vectors

Between 10^5^ and 10^6^ DC were transduced with the different Ad5 vectors separately. For each sample donor, DC were divided into 9 fractions of 100 µl each of serum-free RPMI-1640. They were transduced at 25 pfu of Ad5 vector/cell and incubated for 1 hour at 37°C. For some experiments, replication defective Ad5 with no insert (Ad5-empty) was used at 2500 vp/cell (kindly donated by Dan Barouch, MIT and Harvard, Boston, USA). Cells were then harvested and resuspended at a final concentration of 10^5^ cells/ml in culture medium (RPMI-1640 supplemented with 5% pooled human AB serum [Sigma Aldrich, UK], 1% penicillin, 1% streptomycin and 1% L-glutamine [PAA Laboratories, UK]). Cells were incubated for 24 hours then matured overnight with bacterial lipoplysacharide (LPS, 1 µg/ml, Sigma Aldrich, UK) and IFN-γ (1000 U/ml, Miltenyi Biotec, Germany). To assess targeting of vaccine genes to the proteasome, DC were transduced with the different Ad5 constructs, cultured in the presence or absence of LPS and IFN-γ for 24 h and then cultured for a further 24 h in the presence or absence of 10 µM of the proteasomal inhibitor MG132 (Sigma Aldrich, UK). The cellular level of transgene protein was inferred by measuring HA expression. DC were stained with anti-HA tag-PE antibody (Abcam, UK) in the presence of 0.5% Saponin (Sigma Aldrich, UK) in PBS. Cells were then fixed with 4% paraformaldehyde (Sigma Aldrich, UK) in PBS and analysed by flow cytometry.

### Isolation of naïve T cells

Autologous naïve T cells were isolated by negative selection using magnetic beads from the high density Percoll fraction. First, total T cells were enriched using the Pan T cell isolation kit II (Miltenyi Biotec, Germany). CD45RO positive memory T cells were subsequently depleted using CD45RO microbeads (Miltenyi Biotec, Germany) according to the manufacturer's guidelines. The purity of isolated cells was found to be greater than 90% as assessed by double staining with antibodies against CCR7 (R&D systems, UK) and CD45RA (BD Biosciences, UK) by flow cytometry.

### *In vitro* priming and expansion of T cells

Antigen-specific naïve T cells were primed and expanded as previously described in [37], [41] with slight modification. Briefly, naïve T cells were co-cultured with autologous Ad5-transduced mature DC at a ratio of 20∶1. Cells were incubated for 7 days, harvested, viability assessed by Trypan blue exclusion (Sigma Aldrich, UK) and resuspended at a concentration of 2×10^6^ cells/ml. Cells were co-cultured with a new batch of autologous Ad5-transduced mature DC at a ratio of 20∶1. This boosting of T cells with DC was repeated at least 2 to 3 times on a weekly basis. In addition, from day 9 post initial DC∶T cell priming (2 days after the 1^st^ DC∶T cell boost) cells were maintained in culture medium supplemented with IL-2 (50 U/ml), IL-7 (10 ng/ml) and IL-15 (2.5 ng/ml)(all from Miltenyi Biotec, Germany). Cells were fed with fresh cytokines every 2–3 days and medium was changed whenever necessary.

### IFN-γ ELISPOT assays

After 3–4 weeks of T cell expansion, cells were harvested, washed twice and rested in cytokine-free culture medium for 48 hours. For some experiments CD4 T cells were depleted using CD4 Dynabeads (Invitrogen, UK) according to the manufacturer's guidelines. Cells were recounted and mixed with autologous PBMC at a 1∶1 ratio in culture medium. 96-well Polyvinylidene fluoride (PVDF) membrane plates (MSIPS4510, Millipore, UK) were activated with 70% ethanol for 4 minutes. Plates were washed 3 times with sterile PBS and coated overnight with mouse anti-human IFN-γ monoclonal antibody (10 µg/ml, clone 1-D1K, Mabtech AB, Sweden) in sterile PBS. Plates were washed then blocked for at least 2 hours with RMPI-1640 containing 10% human AB serum. After further washing cells were added at a concentration of 10^5^ cells/well. One hundred and twenty five individual SIV-mac239 gag overlapping peptides (15-mers overlapping by 11aa, obtained from the NIH AIDS Reference Reagent Program. Sequences of peptides 1–125 are given at https://www.aidsreagent.org/search_reagents.cfm ) were added to wells in duplicate at a concentration of 2.5 µg/ml. For T cells that were expanded with minigenes, only peptides spanning the SIV-gag fragment were used. Negative control wells consisted of cells cultured in the absence of peptides and in the presence of 0.025% dimethyl sulfoxide (DMSO, Sigma Aldrich, UK). Phytohaemagglutinin-stimulated cells (PHA, 10 µg/ml, Sigma Aldrich, UK) served as a positive control. Plates were incubated overnight at 37°C then washed 6 times with PBS. Secondary mouse anti-IFN-γ antibody (1 µg/ml, clone 7-B6-1, Mabtech AB, Sweden) was added to all wells and incubated for 2 hours. Plates were washed and ABC peroxidase-avidin-biotin complex (Vector labs, UK) was added for 1 h at room temperature. Spots were then developed by addition of filtered AEC (Sigma Aldrich, UK) substrate solution for 4 min. and finally plates were washed with water and dried overnight. Spots were read using an automated AID ELSIPOT reader (AutoImmun Diagnostika, Germany).

### Intracellular cytokine staining

DC were transduced with the different Ad5 vectors as previously indicated. Cells were matured overnight with LPS and IFN-γ followed by co-culture with autologous expanded T cells and after 3 h brefeldin A was added and culture continued for a further 15 h. Cells were harvested and surface stained for 20 mins at 4°C with anti-CCR7-PE (R&D Systems, UK), CD3-PE-Cy5, CD45RA-APC, CD8-APC-H7, CD4-HorizonV500 (all from BD Biosciences, UK). Cells were washed and fixed with BD Stabilising fixative (BD Biosciences, UK) for 10 mins, washed and resuspended in 0.5% Saponin (Sigma Aldrich, UK) in PBS (PAA Laboratories, UK). They were then stained for 30 mins at room temperature with anti-IFN-γ-PE-Cy7, IL-2-HorizonV450, and TNF-α-FITC antibodies. Finally, cells were washed and fixed with BD stabilising fixative (BD Biosciences, UK). Cells were acquired using a 3-laser configuration LSRII flow cytometer (BD Biosciences, USA). Cytokine secretion by antigen specific T cells were analysed by FlowJo (Tree Star Inc, USA). The gating strategy for the Identification of multifunctional T cells was performed as previously published in [42].

### Levels and stability of transgene mRNAs for different Ad5 gag constructs

Confluent A549 cells were infected for 24 h with 10 pfu/cell of each Ad5 construct. Cells were then harvested or actinomycin D (8 µg/ml) added and cells incubated at 37°C for a further 3 h before harvesting. mRNA was extracted using the Miltenyi Biotec mRNA isolation kit and treated with DNase prior to reverse transcription to synthesise cDNA employing AMV reverse transcriptase and oligo-dT. Quantitative real time PCR was performed with a Roche 4 LightCycler 480 and the LightCycler 480 Syber Green master kit (Roche, UK) with the primers shown in Table S1. MF4 primers were used to amplify full length gag. The levels of mRNA were normalised to the amount of mRNA to β actin.

### Statistical analysis

Data depicted in the figures are shown as means ± standard deviation using Graphpad Prism 5 (GraphPad Software, San Diego, CA, USA). Statistical evaluations were performed using SPSS 17.0 software (SPSS Inc., Chicago, IL, USA). A one-way ANOVA (repeated-measures) was employed to assess differences between groups. When significance was obtained (p values below 0.05), a Fisher LSD post-hoc test was used to examine pairwise comparisons.

## Results

### Effective transduction of human DC with Ad5 expressing SIV genes

**Figure 1:**
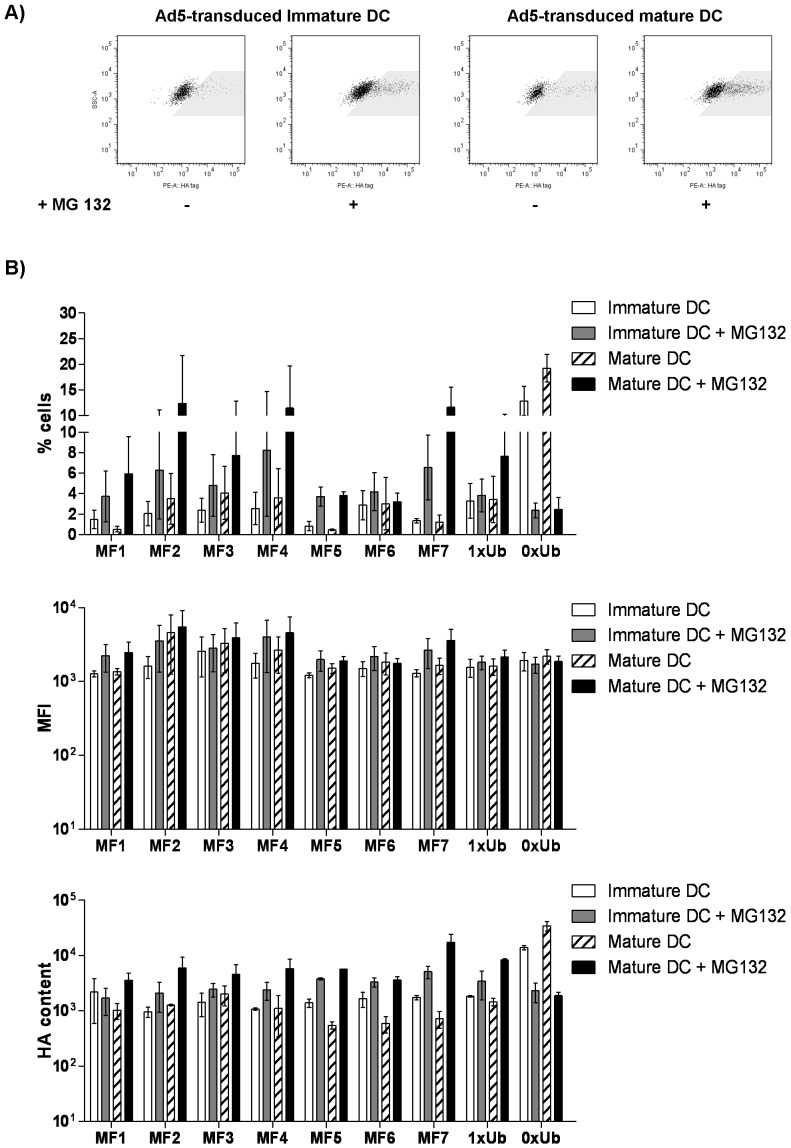
Transduction of DC with Ad5 vectors expressing SIV-gag genes. DC were transduced with Ad5 constructs for 24 hours and cultured in the presence or absence of LPS and IFN-γ for an additional 24 hours. Untransduced DC were used as controls. MG132, a proteosomal inhibitor, was added to half of the samples in the last 16 hours of culture. Expression of HA intracellularily was monitored by flow cytometry. Representative dot plot profiles are shown in **A**) whilst cumulative data is shown in **B**). Percentages shown in B have been corrected for background PE expression in untransduced DC. Total HA content (bottom graphs) were calculated by multiplying the percentages of DC expressing HA by the mean fluorescence intensity of HA in each sample. Bars show mean values from 3 independent samples ± standard deviations.

In order to detect expression of SIV-gag gene fragments within DC, all genes used in this study were tagged at the 3′ prime end with a hemaglutinin (HA) sequence (Figure S1C) and expression measured in the presence or absence of MG132, a proteosomal inhibitor. As shown in Figure 1A, we were able to detect HA intracellularily by flow cytometry using an anti-HA antibody, indicating successful transduction of DC with Ad5-SIV gag constructs. In the absence of the proteasomal inhibitor, the percentages of mature DC expressing HA were relatively low (average 2.49% [range 0.01–9.25%]) when vectors expressing ubiquitinated genes (MF1–MF7 and 1xUb-SIV-gag) were used. While mature DC that were transduced with Ad5 expressing non-ubiquitinated full-length SIV-gag (0xUb-SIV-gag) showed an average of 19.25% (range [15.55–24.49]) cells expressing HA (Figure 1B). In order to verify that ubiquitination of SIV-full-length gag and fragmented genes had effectively increased proteasomal targeting, we blocked the proteasome with the MG132 inhibitor. This resulted in a 2–10 fold increase in the percentages of DC expressing HA when vectors expressing ubiquitinated genes were used (1xUb MF1–MF7 and 1xUb gag, Figure 1B). Similarly, in the same cultures, there was an increase of 4–45 fold in the total content of HA defined as percentages of cells expressing HA multiplied by the mean fluorescence intensity of HA (Figure 1B). Surprisingly, addition of MG132 resulted in a decrease in HA expression when non-ubiquitinated SIV-gag was utilised to transduce DC (Figure 1B).

To ensure responses were not due to major differences in expression or stability of mRNA of different constructs, A549 cells, an epithelial cell line, were transfected with equal infectious units of each construct and after 24 h mRNA was extracted or transcription inhibited by addition of actinmycin D for 3 h prior to mRNA extraction. The levels of mRNA expressed relative to that of β actin were determined for each construct and the relative amount of mRNA for each modified gag construct compared with that for full length unmodified gag. In two independent experiments there were no marked differences in transgene mRNA expression, apart from a somewhat higher expression of MF2, between the different modified gag constructs and that of mRNA for full length gag (Figure S2A). Similarly in transcription blocking experiments there was little difference in the mRNA stability of different constructs (Figure S2B).

### Fragmentation and/or ubiquitination does not alter CD4/CD8 ratios

**Figure 2:**
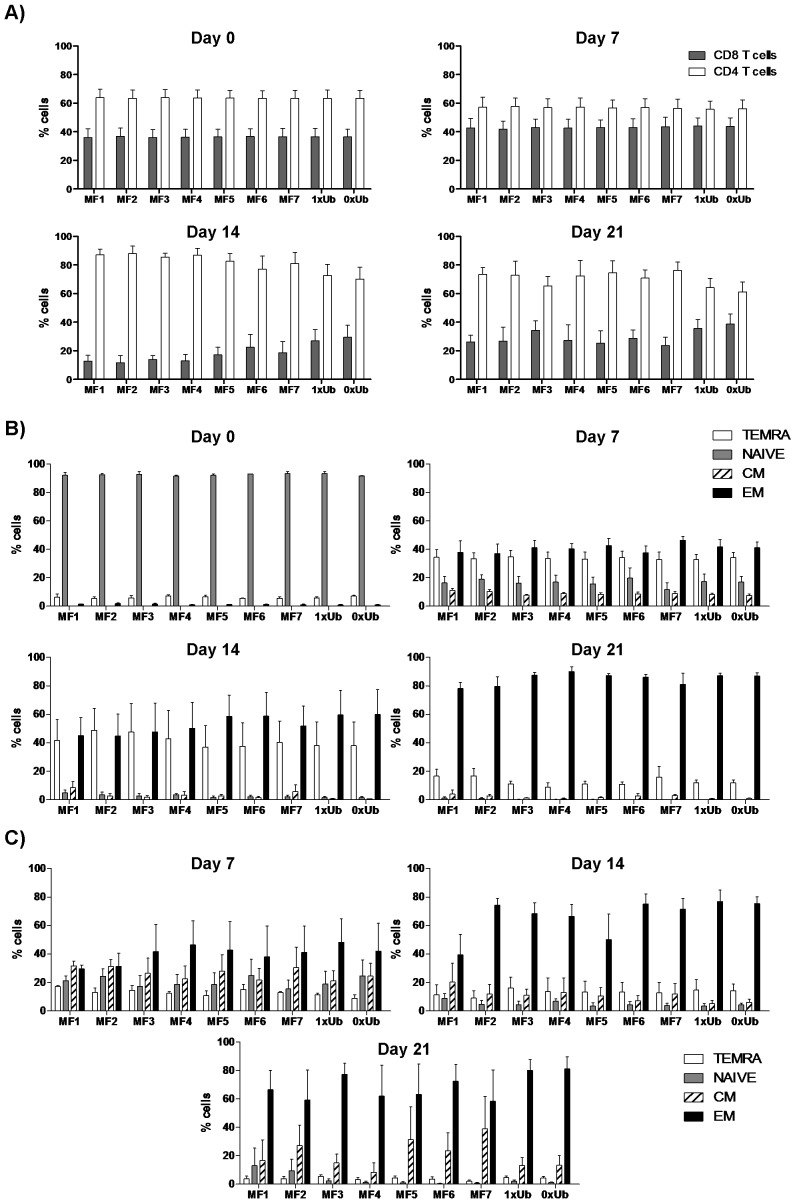
CD4/CD8 T cell proliferation and memory differentiation following *in vitro* prime/boost protocol. Purified naїve T cells were primed and boosted with Ad5-trasnduced DC expressing non-ubiquitinated full-length SIV-gag (0xUb), ubiquitinated full-length SIV-gag (1xUb), or ubiquitinated SIV-gag mini genes (MF1–MF7). A) The percentages of expanded CD3^+^ T lymphocytes expressing CD4 (open bars) or CD8 (Gray bars) are shown on day 0, day 7, day 14 and day 21 post initial DC-T cell priming. B) the proportions of CD3+ CD8+ T cell subsets out of total CD8 T cells that were CCR7+ CD45RA+ (Naїve T cells, gray bars), CCR7− CD45RA+ (Terminal effector cells [TEMRA], white bars), CCR7−CD45RA− (Effector memory [EM], hatched bars), and CCR7+ CD45RA− (Central Memory [CM], closed bars) are shown for days 0, 7, 14 and 21 post initial DC-T cell co-cultures. C) same as B except that data show memory differentiation of cytokine (IFN-γ, IL-2, or TNF-α^+^) CD3^+^ CD8^+^ cells on day 7, 14, and 21 in response to overnight stimulation with transduced mature DC. Bars show mean values out of four samples ± standard deviations.

Isolated naïve T cells were stained for expression of CD4, CD8, CD45RA and CCR7 receptors prior to co-culture with Ad5-transduced autologous DC. Expanded T cells were also analysed for expression of these markers on a weekly basis. As shown in Figure 2A, the percentages of CD8 T cells within the isolated naïve T cell population had a mean of 36% (range [30–42%]) at day 0. All T cell cultures from individual donors expanded at similar rates regardless of the Ad vector utilised (data not shown) and there were no differences between the vectors in terms of CD4/CD8 ratio at each time point suggesting that fusion to ubiquitin had not impaired the generation of CD4 T cells (Figure 2A). However, with all vectors there was a slight preferential expansion of CD8 over CD4 T cells after 7 days of co-culture with DC, followed by a steep contraction in CD8 T cells after 14 days post initial stimulation. A recovery in the percentages of CD8 T cells was then observed on day 21 (Figure 2A) and on day 26 (data not shown).

### Fragmentation and/or ubiquitination does not alter CD8 T cell memory generation

Fusion with ubiquitin would be expected to preferentially target the antigen to the MHC class I pathway, possibly at the expense of the class II pathway, and since CD4 T cells are required for development and maintenance of CD8 T cell memory we asked whether fusion to ubiquitin or fragmentation would influence the CD8 T cell memory phenotype. Expanded CD8 T cells were stained with antibodies against CCR7 and CD45RA to identify the different differentiation stages of T cell memory development. CD8 T cells that were CCR7+ CD45RA+, CCR7− CD45RA+, CCR7−CD45RA−, and CCR7+CD45RA− were defined as naïve, terminal effector (TEMRA), effector memory (EM), and central memory (CM) T cells respectively [43]. We performed this analysis on a weekly basis gating on the total expanded CD8 T cell population (Figure 2B) and on antigen-specific CD8 T cells (Figure 2C). The latter were defined as IFN-γ^+^, IL-2^+^, or TNF-α^+^ CD3^+^ CD8^+^ CD4^−^ live cells in response to stimulation with Ad5-pulsed DC.

For the total naïve CD8 T cell population co-culture with Ad5-SIV-gag-transduced DC resulted in their differentiation into terminal effector and effector memory cells in similar proportions after 7 days (Figure 2B). By day 14, there was an increase in the proportion of effector memory cells, which was more pronounced after 21 days at which time there was a marked decrease in the total proportion of terminal effectors (Figure 2B). At 7 days 5–10% of the total CD8 T cell population had a central memory phenotype but at later time points only minimal numbers were observed. No differences in CD8 T cell phenotype were observed between the different constructs indicating that ubiquitination or genetic fragmentation did not alter the memory differentiation. The majority of cells expanded in the *in vitro* priming system are not antigen specific and thus it is more relevant to analyse the phenotype of the antigen-specific cells as defined by their ability to secrete cytokines upon stimulation. Analysis of cytokine producing CD8 T cells revealed a slightly different picture (Figure 2C). On day 7, less than 20% of cells were single positive for CD45RA (TEMRA) whereas between 20 and 50% of cells belonged to the other three CD8 T cell subsets (Figure 2C). By day 14, the majority of cytokine producing CD8 T cells were effector memory cells, and of note, and in contrast to the whole population by day 21, a mean of 10–35% of cytokine positive antigen specific cells were single positive for CCR7 (central memory cells, Figure 2C). In addition the percentages of central memory cells were more frequent in cultures that were primed and boosted with SIV-gag mini fragments by day 21 (Figure 2C) and day 26 (data not shown).

### Cytokine production and T cell polyfunctionality in response to ubiquitination/fragmentation

**Figure 3:**
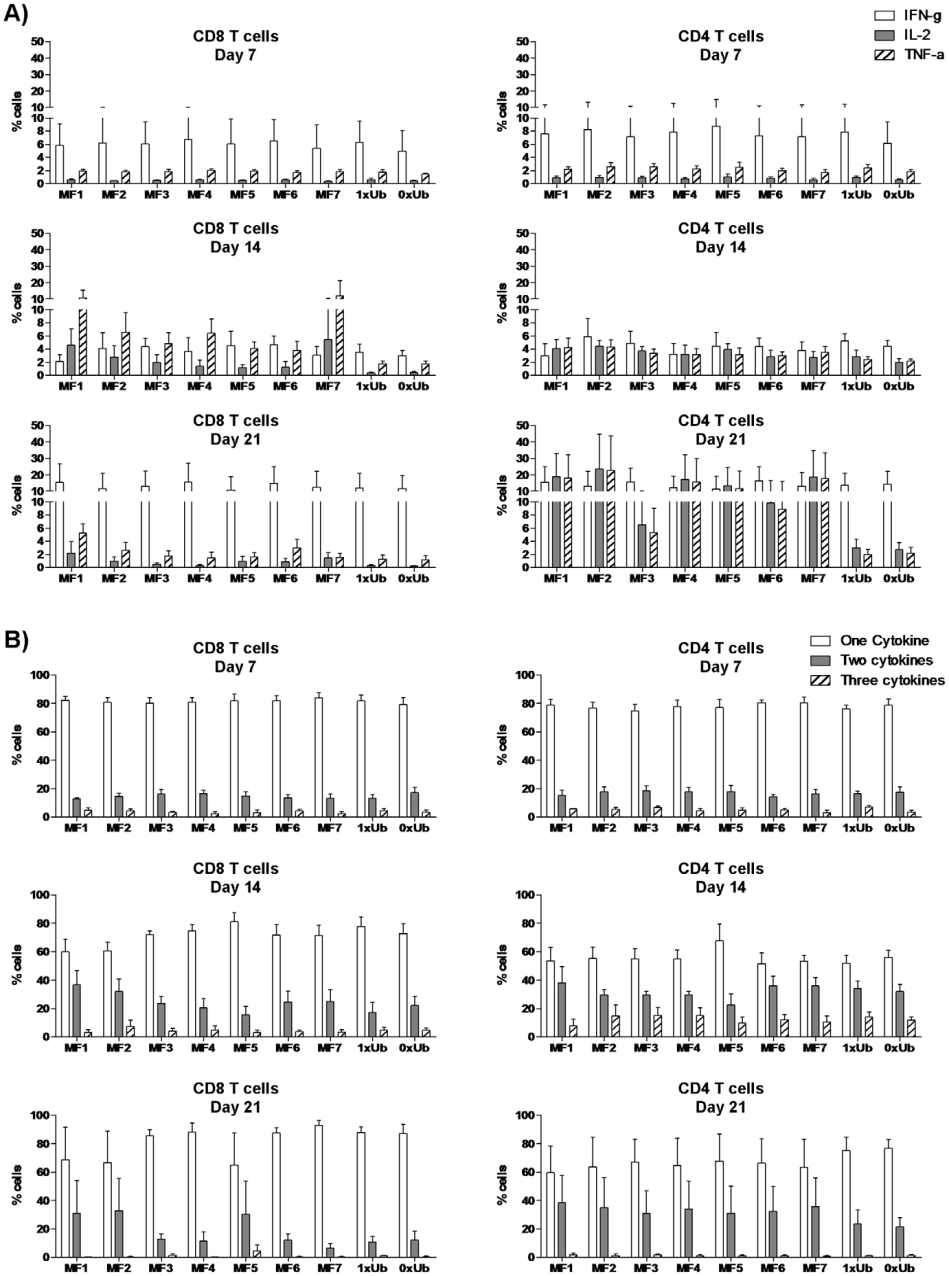
Cytokine production by antigen-specific CD4 and CD8 T cells. Purified naive T cells were primed and boosted weekly with Ad5-trasnduced DC expressing non-ubiquitinated full-length SIV-gag (0xUb), ubiquitinated full-length SIV-gag (1xUb), or ubiquitinated SIV-gag mini genes (MF1–MF7). T Cells were restimulated overnight with Ad5-trasnduced mature DC expressing the respective genes on days 6, 13, and 20 post initial DC-T cell priming. The percentages of IFN-γ (open bars), IL-2 (Gray bars), and TNF-α (hatched bars) producing CD8 (left panels) and CD4 T cells (right panels) are shown in A). The proportions of CD8 (left panels) and CD4 T cells (right panels) that were producing a single cytokine (open bars), two cytokines (gray bars), or all three cytokines (hatched bars) out of the total population of cytokine producing cells are shown in B). For both A and B, bars represent mean values out of four samples whilst error bars represent standard deviations.

We also addressed the question of whether ubiquitination or gene fragmentation would alter the cytokine profiles of memory CD4 and CD8 T cells. T cells were primed and expanded as previously indicated using the different Ad5 vectors. IFN-γ, IL-2 and TNF-α production by CD4 and CD8 T cells was measured by intracellular cytokine staining on a weekly basis in response to overnight culture with DC expressing the respective SIV gag genes. As illustrated in Figure 3A, on day 7, a mean of 5% CD8 T cells and 7% CD4 T cells secreted IFN-γ regardless of which vectors were used. On day 14, the cytokine profile of CD8 T cells shifted from IFN-γ to a TNF-α positive profile, which was more pronounced in cultures expanded using fragmented SIV gag (Figure 3A). This was accompanied with an increase in the percentages of IL-2 secreting CD8 T cells that were expanded using fragmented SIV-gag. CD4 T cells after 14 days of expansion produced equal amounts of IFN-γ, IL-2, and TNF-α and no differences were observed amongst the vectors. The majority of antigen specific CD8 T cells after 21 days of culture secreted IFN-γ (Figure 3A). On the other hand, equal percentages of CD4 T cells produced IFN-γ, IL-2, or TNF-α in response to fragmented SIV-gag whilst IFN-γ was the main cytokine secreted by CD4 T cells that were primed and expanded using constructs expressing full-length SIV gag (Figure 3A).

We also addressed the polyfunctionality of these responses weekly. The majority of antigen-specific CD4 and CD8 T cells produced a single cytokine (around 80% of the response, Figure 3B) after 7 days of culture and less than 10% of cells were positive for two or more cytokines. By day 14, more polyfunctional CD4 and CD8 T cells appeared averaging around 30% of cells secreting two cytokines (Figure 3B), and a mean of 12% (range [0–30%]) cells producing three cytokines in the case of CD4 T cells. By day 21, the majority of antigen specific CD4 and CD8 T cells were producing either one or two cytokines but not all three (Figure 3B). At this stage, no significant differences were observed between the different constructs utilised.

### Adenovirus specific responses generated *in vitro*

Although no major differences were observed between the different constructs utilised in terms of T cell memory differentiation or cytokine production, it is probable that these responses include Ad5-specific T cells which may obscure SIV-specific T cell responses. To address this issue, we repeated T cell priming and expansion using DC that were transduced with either Ad5 with no insert (Ad5-empty) or Ad5 expressing 0xUb-gag using cells from four donors. We monitored T cell memory differentiation and cytokine production over a four week period (Figures S3 and S4). No differences were observed in CD4/CD8 T cell ratios between cultures primed with Ad5-empty or Ad5-0xUb gag (Figure S3A) or T cell memory differentiation over time (Figure S3B). This may merely reflect the nature of this in vitro system in that the cytokines used (IL-2, IL-7, and IL-15) with repeated DC stimulations may dictate the type of memory responses generated regardless of the antigen used.

The specificity of T cells that were primed and expanded against Ad5-empty or Ad5-0xUb-gag were assessed on a weekly basis by intracellular cytokine staining (Figure S4). For these experiments, a sample of expanded T cells were collected weekly and re-stimulated overnight with DC that were transduced with Ad5 empty or Ad5-gag-0xUb and intracellular cytokines were then measured in both CD4 and CD8 T cells. Surprisingly, T cells that were expanded against Ad5-0xUb-gag produced similar or higher levels of IFN-γ, IL-2, and TNF-α when challenged with DC that were transduced with Ad5 empty compared to Ad5-0xUb-gag-transduced DC. Similarly, Ad5-empty-specific T cells produced similar or lower levels of cytokines when they were re-stimulated with Ad5-0xUb-gag compared to Ad5-empty-transduced DC. Thus, this may suggest that DC expressing SIV-gag genes were less potent at presenting antigen compared to those transduced with Ad5-empty.

### Gene fragmentation but not ubiquitination increases the breadth of SIV-gag-specific T cells

**Figure 4:**
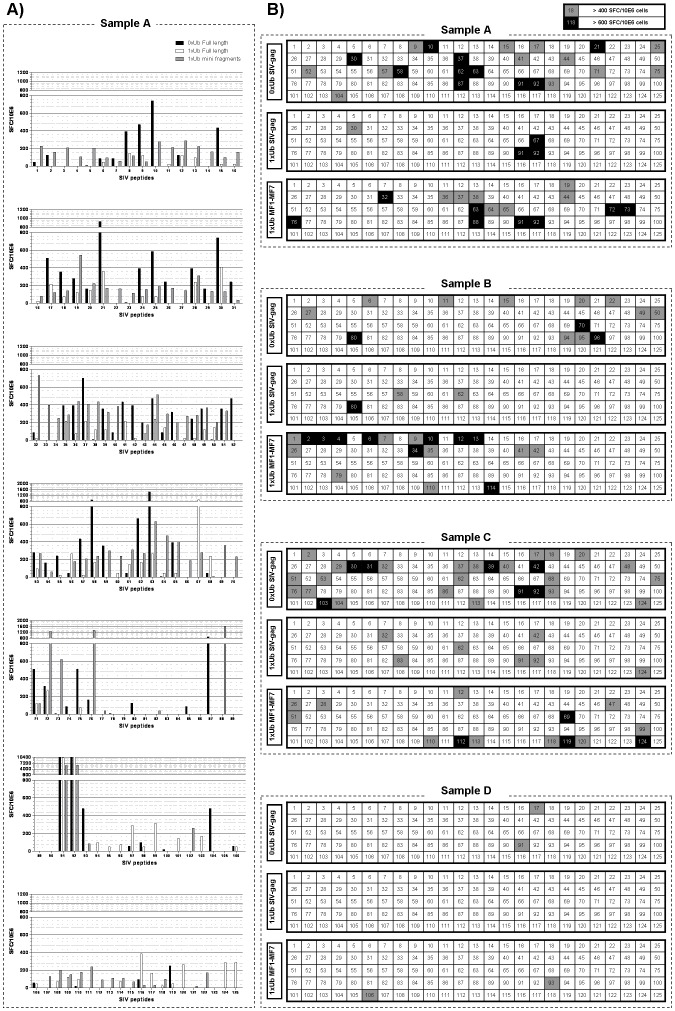
Fragmentation augments SIV-gag specific T cell responses. Purified naive T cells were primed and boosted weekly with Ad5-transduced DC expressing non-ubiquitinated full-length SIV-gag (0xUb), ubiquitinated full-length SIV-gag (1xUb), or ubiquitinated SIV-gag mini genes (MF1–MF7) separately. SIV-specific T cell responses against 125 overlapping SIV-gag peptides were measured using IFN-γ ELISPOT assays on day 21 post initial DC-T cell priming. Spot forming cells (SFC) per one million T cells in cultures that were initially stimulated with 0xUb (closed bars), 1xUb (open bars), or SIV-gag mini fragments (gray bars) are shown for a representative sample in the left panel, where each graph from top to bottom shows responses against individual SIV peptides spanning the mini-fragment regions MF1 to MF7. The data from 4 independent samples are summarised in the right hand side tables where strong responses (>600 SFC/10^6^ cells) are shown in black and medium responses (>400 SFC/10^6^ cells) are highlighted in gray. All data were corrected for background IFN-γ production in response to mock stimulations. Note that sample A represents CD8 specific T cell responses whereas samples B, C, D represent responses using a mixture of CD4 and CD8 T cells. Sequences of all 125 peptides numbered as in these experiments may be found at https://www.aidsreagent.org/search_reagents.cfm.

Since we were unable to distinguish SIV-specific responses from those generated against the Ad5 vector using intracellular cytokine staining, we assessed the specificity and breadth of responses against SIV transgenes from the individual vectors by performing ELISPOT assays using individual peptides (15 mers overlapping by 11 aa) spanning the entirety of SIV-mac239 gag were used to stimulate the expanded T cells in IFN-γ ELISPOT assays (Figure 4). A representative sample showing the number of spot forming cells (SFC) per million cells against each peptide is shown in Figure 4A where all responses were corrected for background secretion of IFN-γ in response to peptide-free DC. For simplicity, the responses generated in all samples are summarised in Figure 4B, where only responses higher than 400 SFC per million cells are shown. The choice of this stringent cut off was based on a minimum of 4 times standard deviation of background responses. The mean background ± standard deviation for all experiments was 228±89 SFC per million cells. Responses that were higher than 600 SFC were deemed strong responses and are also highlighted in Figure 4B.

Overall, there was a mean frequency of 16.5 peptides (range 2–29 peptides) recognised by cells expanded using vectors expressing non-ubiquitinated full-length SIV-gag (Table 1). In contrast, priming and expansion of T cells using ubiquitinated full-length SIV gag resulted in responses against a mean of only 3.5 peptides (range 0–7 peptides), 75% of them being recognised in the unmodified full-length gag (Table 2). These results indicate that ubiquitination of SIV-gag resulted in a loss in the frequency of epitopes recognised though this fell just short of statistical significance (p = 0.06, Table 1). Conversely, Ad5 vectors expressing ubiquitinated fragmented SIV-gag genes induced responses against a mean of 12.25 peptides (range 2–18) compared to a mean of 3.5 peptides (range 0–7 peptides) for full length ubiquitinated gag (Table 1). In contrast to the ubiquitinated full-length gag only 13% of the peptides were also recognised in the unmodified full-length SIV-gag and only 4% were identical to stimulation with the ubiquitinated full-length form (Table 2). Since all mini genes were preceded with a ubiquitin sequence, the effect of fragmentation on the T cell repertoire was deduced by comparing the mini fragments to the ubiquitinated full-length SIV gag. In this is case, fragmentation induced a statistically significant increase in the frequency of recognised epitopes and a diverse T cell repertoire (p = 0.05, Tables 1 and 2). Thus ubiquitinated gag fragments induced responses to a greater number of epitopes than observed with ubiquitinated full length gag and responses to epitopes that were not induced by full length unmodified gag.

**Table 1:** The breadth of T cell responses generated using ubiquitin/fragmented genes.

	Number of peptides recognised (>400 SFC/10E6 cells)						Number of Strong positive responses (>600 SFC /10E6 cells)					
**Donor ID**	0xUb		1xUb		MF		0xUb		1xUb		MF	
**Sample A**	22		4		15		10		3		8	
**Sample B**	13		3		18		3		1		9	
**Sample C**	29		7		14		7		0		4	
**Sample D**	2		0		2		0		0		0	
**Median**	**17.5**		**3.5**		**14.5**		**5**		**0.5**		**6**	
**Mean**	**16.5**		**3.5**		**12.25**		**5**		**1**		**5.25**	
**minimum**	**2**		**0**		**2**		**0**		**0**		**0**	
**maximum**	**29**		**7**		**18**		**10**		**3**		**9**	
**comparison with**	**1xUb**	**MF**	**0xUb**	**MF**	**0xUb**	**1xUb**	**1xUb**	**MF**	**0xUb**	**MF**	**0xUb**	**1xUb**
**P values**	**0.06**	**0.4**	**0.06**	**0.05** *	**0.4**	**0.05** *	**0.11**	**0.9**	**0.11**	**0.08**	**0.9**	**0.08**

* Indicates statistically significant differences

**Table 2:** Diversity of the T cell responses generated using ubiquitin/fragmented genes.

	Percentage Similarity (%)*					
Construct A	0xUb		1xUb		MF	
Compared with Construct B	1xUb	MF	0xUb	MF	0xUb	1xUb
**Sample A**	13.64	22.73	75	50	33.33	13.33
**Sample B**	7.69	7.69	33.33	0	5.56	0
**Sample C**	20.69	10.34	85.71	14.29	21.43	7.14
**Sample D**	0	0	n.a.	n.a.	0	0
**Median**	**10.66**	**9.02**	**75**	**14.29**	**13.49**	**3.57**
**Mean**	**10.5**	**10.19**	**64.68**	**21.43**	**15.08**	**5.12**

* The percentage similarity amongst the constructs used in this study was calculated as follow: the number of common peptides that are recognised by both construct A and B divided by the total number of peptides recognised by construct A multiplied by 100. n.a. not applicable.

## Discussion

Data presented in this study addresses two different strategies aimed at broadening T cell responses to codon optimised SIV gag, namely fragmentation and fusion with ubiquitin. A marked increase in the number of epitopes recognised in ubiquitin-fused mini genes compared to ubiquitin-fused full-length gag was observed. Thus gene fragmentation is a potential strategy for improving the breadth of HIV vaccines. Since each gag fragment was fused to ubiquitin the appropriate comparison for assessing the response to fragmentation is with full length gag fused to ubiquitin. However, since fusion of ubiquitin to full length gag greatly reduced responses compared to unmodified full length gag the response to gag fragments that have not been fused to ubiquitin needs to be investigated. To ensure that DC were not transfected with more than one construct, thus potentially increasing competition which fragmentation aimed to reduce, separate cultures transfected with single constructs were performed. For vaccination it would be desirable to deliver different gene fragments to different sites to avoid DC being transduced with more than one fragment. Although the results of the fragmentation experiments were encouraging it is possible that in vitro results may not mirror responses in vivo and vaccination studies are needed to confirm that fragmentation of gag is advantageous. In contrast we found that fusion of gag to ubiquitin reduced the breadth of the T cell response. Fusion of ubiquitin to a number of different pathogen genes including influenza NP [31], LCMV NP [32], HIV nef [29], [30] and some but not other malarial antigens [44] has been reported to enhance immunogenicity. We found that fusion of ubiquitin to SIV gag resulted in a lower T cell responses both in magnitude and breadth. Interestingly, Fluet and colleagues demonstrated in a murine study that vaccination with the matrix capsid portion of SIV-gag fused at the N-terminus to ubiquitin resulted in a 2–3 fold reduction in the magnitude of T cell responses against SIV-peptide pools compared to unmodified SIV-gag [45]. Similarly, an earlier study by Wong et al [46] did not show enhanced responses in mice vaccinated with vectors containing unstable gag constructs.

We anticipated that increased degradation of protein via the ubiquitin-proteasome (UPS) pathway (reviewed in [47]) would preferentially direct antigen through the MHC class I pathway at the expence of the class II pathway leading to higher proliferation of CD8 T cell over CD4 T cells, a hypothesis similar to that proposed by Wong et al [46]. This however was not the case in our system, where the percentages of expanded CD4 and CD8 T cells over time were comparable. However, it is possible that the addition of IL-2, IL-7 and IL-15 throughout the *in vitro* culture period may have influenced the differentiation of naïve T cells towards effector and central memory cells ([48] and references therein). Although there was higher targeting of SIV-gag to the proteasome using ubiquitinated constructs, which would be expected to increase peptide density on DC surface [33], [49], neither ubiquitination nor fragmentation altered the memory differentiation profiles of expanded CD8 T cells. Notably, responses generated against Ad5-empty resulted in a similar memory profile over time suggesting that the experimental setup of this in vitro assay may be the main driving factor for CD8 T cell differentiation.

Our homologous prime/boost strategy was found to induce T cell responses against the Ad5 vector, responses to the inserts were also induced as indicated by IFN-γ ELISPOT against SIV-gag peptides. With the exception of sample A (Figure 4), which represents antigen specific CD8 T cell responses, insufficient numbers of cells were generated to deplete CD4 T cells from the expanded cells prior to conduction of the ELISPOT assays. Nonetheless, the observation that ubiquitination of SIV-gag resulted in markedly fewer responses compared to its non-ubiquitinated counterpart was consistent throughout the samples. Fragmentation dramatically increased the number of peptides recognised in all samples tested indicating that total T cell responses, and specifically CD8 T cell responses in sample A, were broadened. Though, these responses remained similar or lower than those observed using non-ubiquitinated full length gag.

As fusion with ubiquitin to full-length gag severely reduced the breadth of the response, it is important that the effect of fragmentation is assessed by comparison with ubiquitin-fused full-length gag rather than with unmodified gag. If fragments without the ubiquitin sequence were available and tested, an even broader response may have been observed. Of particular interest was the observation that fragmentation induced many responses against peptides that were not induced by priming with unmodified full-length gag. This may suggest differential antigenic processing induced by fragmentation. A key question is whether these new epitopes whose recognition is stimulated by vectors containing fragmented SIV gag would be expressed by SIV-infected cells *in vivo*. Of relevance here are the requirements for inducing a primary T cell response compared to a secondary response. The requirements for a primary response are more stringent than for restimulation of memory cells where it has been proposed that a memory CD8 T cell could be activated by a single MHC class I peptide complex [50]. Thus if these new epitopes are expressed at too low a concentration in natural infection to induce a primary response there may be a sufficient amount to activate killing by memory CD8 T cells.

Numerous reports in the literature have shown that ubiquitination of genes induces improved antigen degradation which is translated into increased T cell activation, either in clonal size or avidity of response depending on the conditions of the assays [29]–[34], [44]. The observation that despite proteasomal targeting ubiquitination of SIV gag genes in our study resulted in loss or narrowing of T cell responses compared to wild type SIV gag is puzzling. Although we cannot rule out the possibility that this reduction may reflect our in vitro system, in vivo murine studies have shown similar results [45], [46]. Interestingly we have also found that responses to HIV gag fused to ubiquitin are also reduced (Herath et al unpublished data). Notably, T cells were stimulated on a weekly basis and it remains plausible that shorter stimulation intervals may influence the outcome of ubiquitination. The specificity of T cell responses were identified in this study on the basis of secretion of IFN-γ alone (Figure 4) and it is likely that these may not be fully representative of all T cell responses as indicated by intracellular cytokine staining for TNF-α and IL-2 (Figure 3). In addition, since we were unable to quantify the number of expanded CTL by tetramer staining or other means, it is difficult to rule out the possibility that ubiquitination may have in fact resulted in expansion of SIV-gag-specific CD8 T cells that do not secrete IFN-γ, IL-2, or TNF-α and are maybe only positive for perforin and granzymes or perhaps neither of these. Nonetheless, CD8 T cells generated in this study were found to produce mainly IFN-γ by day 21 which is when the ELISPOT assays were carried out. In addition, most clinical trials rely on IFN-γ ELISPOTS to determine the immunogenicity of vaccine candidates in the case of HIV and SIV studies [51].

Genetic fragmentation has previously been suggested as a strategy to overcome antigenic competition and widen the breadth of the T cell response. Employing a similar gag fragmentation strategy to that described here to vaccinate mice, broader immune responses have been induced [34], [35]. Here we show that fragmentation is effective at broadening the immune response in a human system. In both studies of Liu et al [35] and Singh and Barry [34] the fragmented genes were administered as a mixture in a single injection site. Although physically separated, the vectors containing different gene fragments may have been picked up and presented to T cells by the same DC, which may therefore hinder the effects of fragmentation. Thus future *in vivo* studies should consider vaccination regimens where minigenes are administered at different anatomical sites to maximise the effects of fragmentation.

## Supporting Information

Figure S1 **Schematic representation of the experimental plan and reagents utilised.** A) *In vitro* protocol for the generation of SIV-specific memory T cells. DC were generated by culture of monocytes with GM-CSF and IL-4 for 7 days. DC were transduced with Ad5 vectors expressing SIV-gag genes for 24 hours and matured with LPS and IFN-γ for a further 24 hours. DC were co-cultured with purified naїve T cells at a DC∶T cell ratio of 1∶10 for 7 days and boosted on a weekly basis with autologous Ad5-transduced mature DC. Intracellular cytokine staining (ICS) was performed as indicated in Materials and Methods one day following each boost. ELISPOT assays was performed on day 23 post initial T cell priming following a period of at least 48 hours of resting in cytokine-free culture medium. B) Schematic representation of the strategy utilised to fragment SIV-gag. The sequence of SIV-mac251 was divided into 7 segments (MF1-MF7) of similar length (69–92 aa) overlapping by 10 aa. As indicated p17 comprised MF1 and MF2, p24 was divided into three segments (MF3–MF5), p2 and p7 sequences were joined in MF6, while MF7 comprised p1 and p6. C) Schematic representation of Ad5 construct gene inserts. 9 Ad5 vectors were generated to conduct the current investigation. 1 vector expressed full-length SIV gag gene under the influence of a CMV promoter (0xUb). A similar construct expressed the same SIV gag gene with a ubiquitin sequence fused at the amino terminus (1xUb). 7 other Ad5 constructs expressed 7 overlapping SIV-gag gene fragments (as shown in B) fused with a ubiquitin sequence. All genes were tagged with an HA sequence to monitor expression of Ad5 inserts.
(TIF)

Figure S2 **Level and stability of mRNA for modified gag transgenes.** A) The relative expression of different modified gag gene mRNAs in A549 cells as a percentage of mRNA for full length gag. B) The stability of mRNAs from full length unmodified gag and modified gag constructs. The data is presented as the percentage of relative expression to β actin prior to the addition of actinomycin D. Data from 2 independent experiments are shown.
(TIF)

Figure S3 **T cell proliferation and memory differentiation in response to Ad5-empty.** Purified naїve T cells were primed and boosted with DC transduced with either Ad5-empty or Ad5 expressing non-ubiquitinated full-length SIV-gag (0xUb). A) The percentages of expanded CD3^+^ T lymphocytes expressing CD4 (open bars) or CD8 (gray bars) are shown on day 0, day 14, day 21 and day 28 post initial DC-T cell priming. B) the proportions of CD3+ CD8+ T cell subsets out of total CD8 T cells that were CCR7+ CD45RA+ (Naїve T cells, gray bars), CCR7− CD45RA+ (Terminal effector cells [TEMRA], white bars), CCR7−CD45RA− (Effector memory [EM], hatched bars), and CCR7+ CD45RA− (Central Memory [CM], closed bars) are shown for days 0, 14, 21 and 28 post initial DC-T cell co-cultures (N = 4).
(TIF)

Figure S4 **Cytokine production by Ad5-specific CD4 and CD8 T cells.** Purified naive T cells were primed and boosted weekly with DC that were transduced with Ad5-empty (white bars) or Ad5 expressing non-ubiquitinated full-length SIV-gag (0xUb, grey bars). T cells were restimulated overnight with Ad5-empty-transduced mature DC (open bars) or Ad5-0xUb-transduced mature DC (hatched bars). The percentages of IFN-γ, IL-2, and TNF-α producing CD8 (left panels) and CD4 T cells (right panels) are shown after 2, 3, and 4 weeks post initial T cell priming. Bars represent mean values out of four samples whilst error bars represent standard deviations.
(TIF)

Table S1 **Primer sequences for real time PCR.**
(DOCX)
