# Supplementary figures and images for: Fragmentation of SIV-gag Vaccine Induces Broader T Cell Responses
Source: PLoS One. 2012 Oct 31;7(10):e48038. doi: 10.1371/journal.pone.0048038 (PMC3485275; doi:10.1371/journal.pone.0048038)

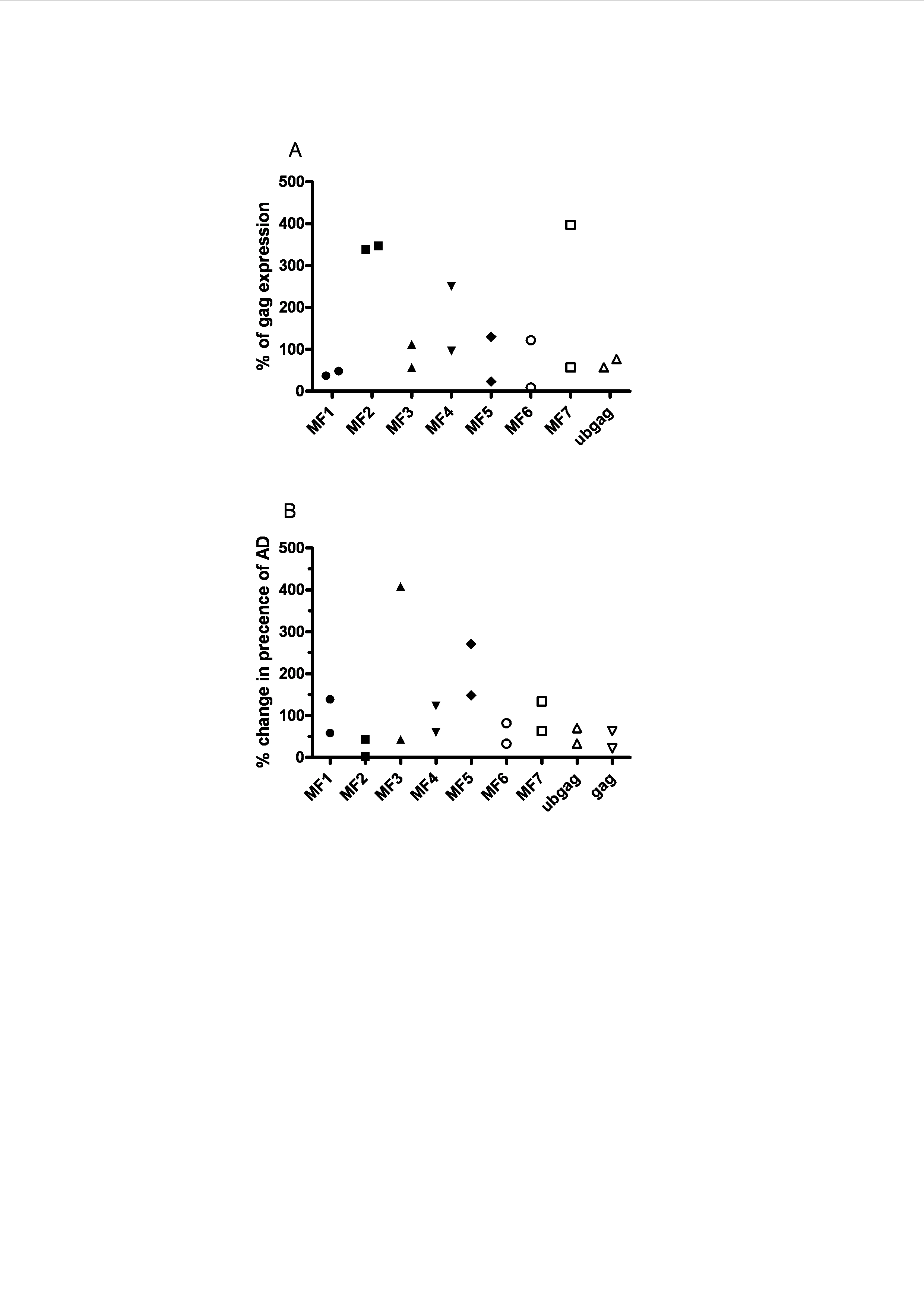

Supplement: Figure S2 — Level and stability of mRNA for modified gag transgenes. A) The relative expression of different modified gag gene mRNAs in A549 cells as a percentage of mRNA for full length gag. B) The stability of mRNAs from full length unmodified gag and modified gag constructs. The data is presented as the percentage of relative expression to β actin prior to the addition of actinomycin D. Data from 2 independent experiments are shown. (TIF) [file pone.0048038.s002.tif]

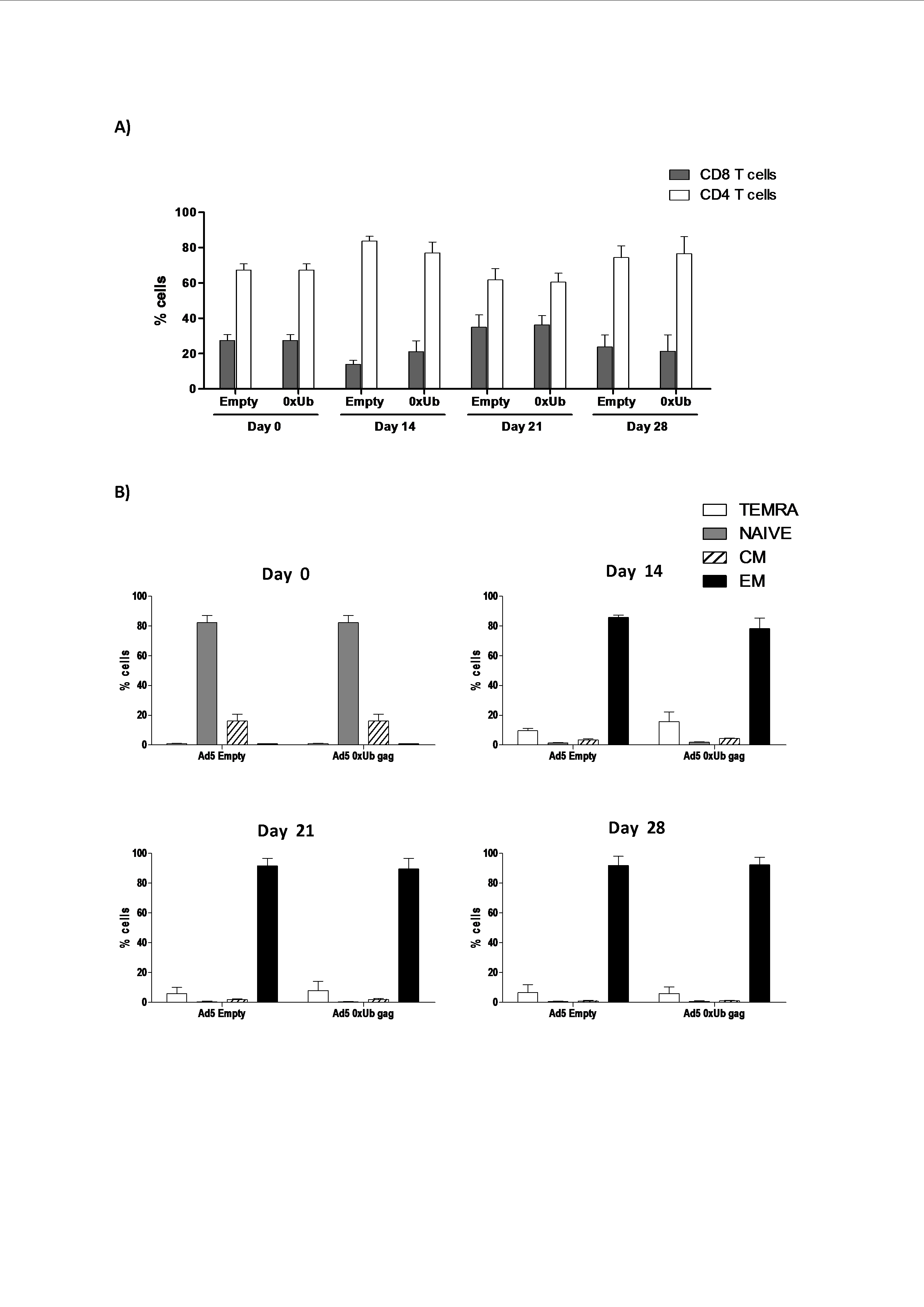

Supplement: Figure S3 — T cell proliferation and memory differentiation in response to Ad5-empty. Purified naїve T cells were primed and boosted with DC transduced with either Ad5-empty or Ad5 expressing non-ubiquitinated full-length SIV-gag (0xUb). A) The percentages of expanded CD3+ T lymphocytes expressing CD4 (open bars) or CD8 (gray bars) are shown on day 0, day 14, day 21 and day 28 post initial DC-T cell priming. B) the proportions of CD3+ CD8+ T cell subsets out of total CD8 T cells that were CCR7+ CD45RA+ (Naїve T cells, gray bars), CCR7− CD45RA+ (Terminal effector cells [TEMRA], white bars), CCR7−CD45RA− (Effector memory [EM], hatched bars), and CCR7+ CD45RA− (Central Memory [CM], closed bars) are shown for days 0, 14, 21 and 28 post initial DC-T cell co-cultures (N = 4). (TIF) [file pone.0048038.s003.tif]

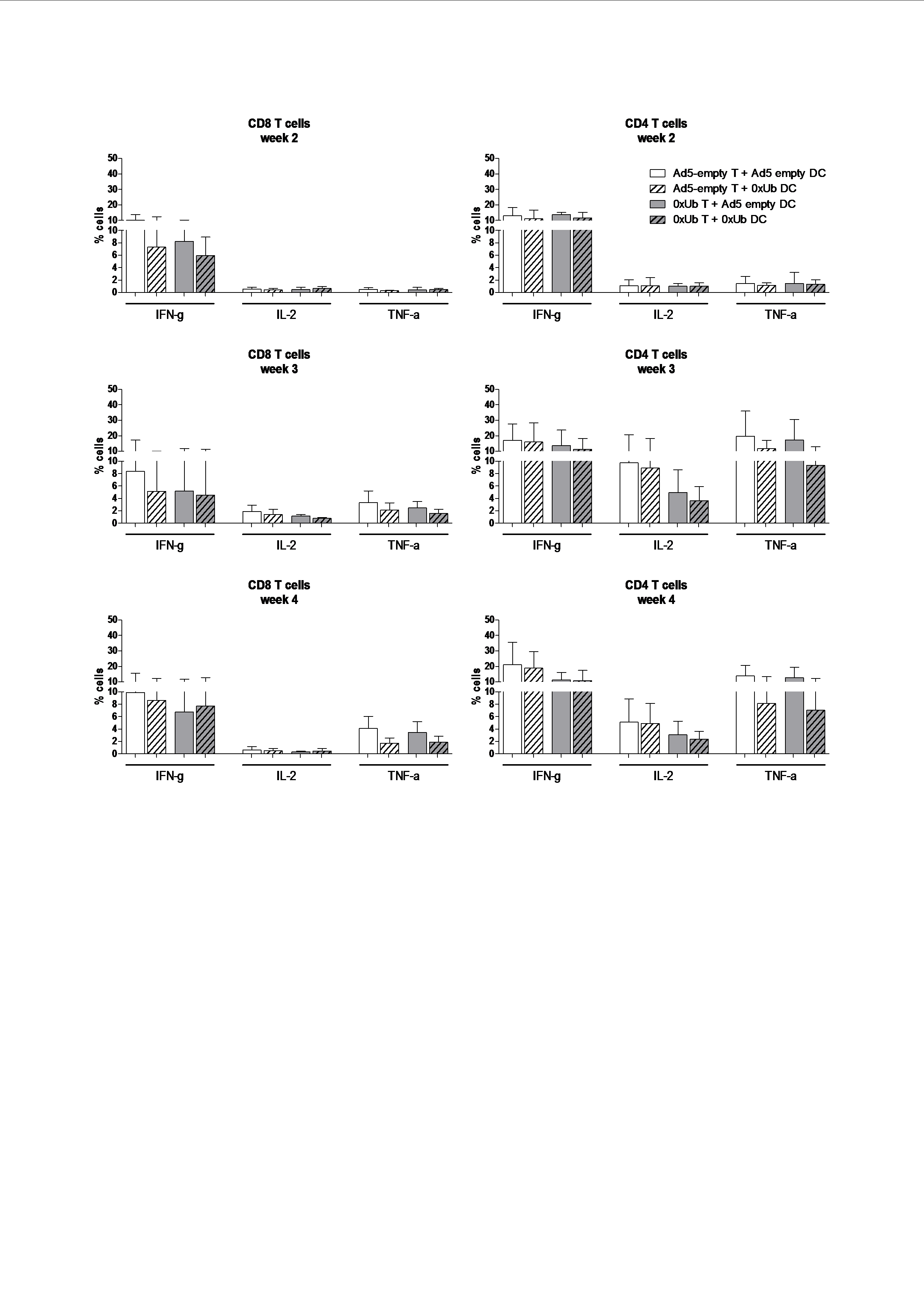

Supplement: Figure S4 — Cytokine production by Ad5-specific CD4 and CD8 T cells. Purified naive T cells were primed and boosted weekly with DC that were transduced with Ad5-empty (white bars) or Ad5 expressing non-ubiquitinated full-length SIV-gag (0xUb, grey bars). T cells were restimulated overnight with Ad5-empty-transduced mature DC (open bars) or Ad5-0xUb-transduced mature DC (hatched bars). The percentages of IFN-γ, IL-2, and TNF-α producing CD8 (left panels) and CD4 T cells (right panels) are shown after 2, 3, and 4 weeks post initial T cell priming. Bars represent mean values out of four samples whilst error bars represent standard deviations. (TIF) [file pone.0048038.s004.tif]
