# Supplementary material for: Fragmentation of SIV-gag Vaccine Induces Broader T Cell Responses
Source: PLoS One. 2012 Oct 31;7(10):e48038. doi: 10.1371/journal.pone.0048038 (PMC3485275; doi:10.1371/journal.pone.0048038)
Supplement: Table S1 — Primer sequences for real time PCR. (DOCX) [file pone.0048038.s005.docx]

| **Primer** | **sequence** |
| --- | --- |
| SIV Mf1 for | GTCCGGCAAGAAGGCCGACG |
| SIV mF1 rev | AGCTCATTGGCGGCCCACAC |
| SIV Mf2 for | CTGGTGCATCCACGCCGAG |
| SIV mF2 rev | AATTGCCGCCTCTTGCCGCTG |
| SIV Mf3 for | AGTTTGGCGCCGAGGTCGTG |
| SIV mF3 rev | TGCAGATCCCAATCGGCGGC |
| SIV Mf4 for | CCCACAGCCTGCACCTCAGC |
| SIV mF4 rev | GGGCCCTGCTTCACATCCAGG |
| SIV Mf5 for | AGCAGACAGATGCCGCCGTG |
| SIV mF5 rev | GGCTTTCTGGCCTGGTCCGC |
| SIV Mf6 for | AAGAGGCCCTGGCCCCAGTC |
| SIV mF6 rev | CAGCCCTGCCTTCTTGGGGC |
| SIV Mf7 for | ACAGGCCGGCTTTCTGGGAC |
| SIV mF7 rev | ACGGCTGGGTCCTCTGGTGG |

Table S1. Primer sequences for real time PCR.
